# Autoimmune enteropathy with a CD8^+ ^CD7^- ^T-cell small bowel intraepithelial lymphocytosis: case report and literature review

**DOI:** 10.1186/1471-230X-11-131

**Published:** 2011-11-29

**Authors:** Shrinivas Bishu, Violeta Arsenescu, Eun Y Lee, H David Vargas, Willem JS de Villiers, Razvan Arsenescu

**Affiliations:** 1 Department of Internal Medicine, University of Kentucky Medical Center, 800 Rose Street, Lexington, Kentucky, 40536, USA; 2 Division of Digestive Diseases and Nutrition, University of Kentucky Medical Center, 800 Rose Street, Lexington, Kentucky, 40536, USA; 3 Department of Surgery, University of Kentucky Medical Center, 800 Rose Street, Lexington, Kentucky, 40536, USA; 4 Department of Pathology and Laboratory Medicine, University of Kentucky Medical Center, 800 Rose Street, Lexington, Kentucky, 40536, USA

## Abstract

**Background:**

Adult onset autoimmune enteropathy (AIE) is a rare condition characterized by diarrhea refractory to dietary therapy diagnosed in patients with evidence of autoimmune conditions. Auto-antibodies to gut epithelial cells and other tissues are commonly demonstrated. Despite increasing awareness, the pathogenesis, histologic, immunologic and clinical features of AIE remain uncertain. There remains controversy regarding the diagnostic criteria, the frequency and types of auto-antibodies and associated autoimmune conditions, and the extent and types of histologic and immunologic abnormalities. CD4+ T-cells are thought to at least responsible for this condition; whether other cell types, including B- and other T-cell subsets are involved, are uncertain. We present a unique case of AIE associated with a CD8+CD7- lymphocytosis and review the literature to characterize the histologic and immunologic abnormalities, and the autoantibodies and autoimmune conditions associated with AIE.

**Case Presentation:**

We present a case of immune mediated enteropathy distinguished by the CD8+CD7- intra-epithelial and lamina propria lymphocytosis. Twenty-nine cases of AIE have been reported. The majority of patients had auto-antibodies (typically anti-enterocyte), preferential small bowel involvement, and predominately CD3+ CD4+ infiltrates. Common therapies included steroids or immuno-suppressive agents and clinical response with associated with histologic improvement.

**Conclusions:**

AIE is most often characterized (1) IgG subclass anti-epithelial cell antibodies, (2) preferential small bowel involvement, and (3) CD3+ alphabeta TCR+ infiltrates; there is insufficient evidence to conclude CD4+ T-cells are solely responsible in all cases of AIE.

## Background

Immune enteropathies are heterogeneous group of rare conditions characterized by intractable diarrhea, damage to intestinal epithelia and constituent cells, and villous atrophy. These enteropathies may be associated with primary immune deficiencies (PIDs) such as the immune dysregulation, polyendocrinopathy, enteropathy, X-linked (IPEX) syndrome, common variable immune deficiency and selective IgA deficiency, or may occur in patients with auto-immune phenomena in the absence of PIDs where it is termed auto-immune enteropathy (AIE) [1].

Typically, the diagnosis of immune mediated enteropathy in the setting of PIDs is clear from the clinical features and a pre-existing diagnosis of PID. In contrast, AIE may be diagnosed in patients with no evidence of PIDs, intractable diarrhea refractory to exclusion diets, and no evidence of celiac disease [1].

Cumulatively, immune enteropathies, including AIE, have an unclear pathogenesis. They are all thought to be mediated by immune phenomena, either through direct damage to gut epithelial cells by auto-reactive cells (IPEX) or auto-antibodies (AIE), or from a propensity for bowel infections (IgA deficiency). However, the exact cell types causing damage and the mechanisms of disease remain unclear in many of these conditions.

Herein, we report a patient with an immune mediated enteropathy distinguished from previously reported cases by an unusual CD8^+ ^CD7^- ^IEL in the absence of a PID. We also review the literature to characterize the histologic and immunophenotyic features of AIE, which our patient's enteropathy most closely resembles.

### Case Presentation

The patient was a 28 year old Asian Indian female of non-consanguineous parents who presented with a 3 year history of non-bloody diarrhea with abdominal cramping. Past medical history was significant for hepatitis A. She denied alcohol or tobacco use, had no chronic medical conditions, was not on any medications including gut irritants such as aspirin/non-steroidials and did not have a history of travel prior to onset of symptoms. Her last travel was to India one year prior to presentation, but she did not report any alleviation or exacerbation of her symptos with the travel/dietary/environmental change. She weighed 81 pounds at presentation. Stool cultures were negative for pathogens. Basic labs, including the erythrocyte sedimentation rate and C-reactive protein were normal, but liver function tests (LFTs) were elevated (Table 1). Abdominal computed tomography (CT) revealed hepatic hypodensities and mesenteric adenopathy. Serology was negative for anti-nuclear (ANA) and anti-smooth muscle antibodies as well as antibodies for cytosolic anti-neutrophil cytoplasm (cANCA), liver-kidney microsomes, liver cytosolic and soluble liver-pancreas antigen. However, pANCA was positive (titer: > 1:20) and she had hyper-gammaglobulinemia (IgG: 2358 (630-1580 mg/dl), IgA: 578 (100-400 mg/dl), IgM: 134 (37-247 mg/dl). Serum ceruloplasmin was normal, and serology for hepatitis B and C were negative. Serum B_12_, folate and hematocrit were normal.

**Table 1:** Liver function test and histologic features pre- and post-prednisone

	Pre-prednisone		Post-prednisone	
	
	Presentation	15 months	21 months	26 months
Liver Function Tests				
AST (U/L)	107	43	41	65
ALT (U/L)	95	46	97	82
ALP (U/L)	240	205	110	123
Albumin (g/dL)	2.2	1.5	2.1	3.5
Histology				

Histology	IEL Intact villi Chronic inflammation	Intact villi Duodenum + jejunum+ illeum: *lymphoplastic infiltrate* Colon + cecum + rectum: *lymphoplastic infiltrate*	IEL Subtotal villous atrophy	IEL Subtotal villous atrophy Segmental areas normal
Immuno-phenotype	not obtained	Jejunum + illeum: *CD3^+^CD5^+^CD8^+^CD7^-^preponderance*	Jejunum: *Polyclonal αβTCR^+^CD3^+ ^preponderance* *CD8^+^CD25^-^CD7^- ^(70% of total IEL)*	Jejunum: *Polyclonal αβTCR^+^CD3^+ ^(94% of total IEL) CD8^+^CD25^-^CD7^- ^(41% of CD3^+ ^fraction)*

**Figure 1:**
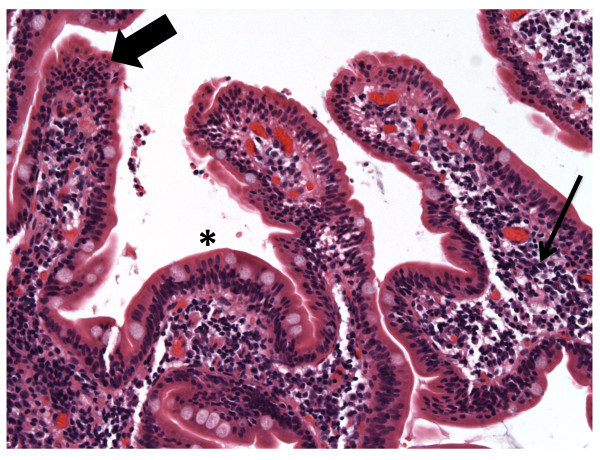
Duodenal biopsy at presentation demonstrating regions of IEL (large arrow) with normal appearing LP (small arrow) and interspersed areas of villous blunting (*).

Histologic examination of intestinal mucosa demonstrated chronic inflammation with duodenal intraepithelial cell lymphocytosis (IEL) (Table 1) (Figure 1). Celiac disease and tropical sprue were therefore considered based on the clinical features and IEL. However, tissue transglutaminase (TTG) IgA was normal (5.3, reference: < 7.0 AU), as were anti-enterocyte antibodies (only IgA tested) (both measured by enzyme linked immuno-absorbant assay of serum). Indirect immunofluorescence on human intestinal tissue samples was not performed. The patient refused liver biopsy, but permitted CT guided biopsies of the enlarged retroperitoneal and periportal nodes; histologic analysis only demonstrated normal stroma. She was diagnosed with auto-immune hepatitis (AIH) based on her revised original International AIH group score of 12 (indicating probable AIH by criteria)[2].

**Figure 2:**
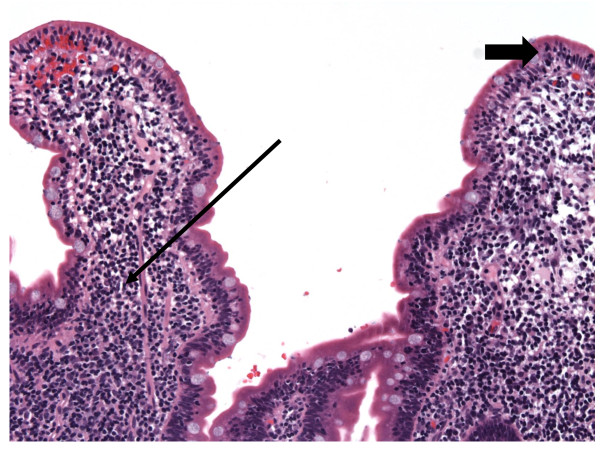
**Duodenal biopsy before steroid therapy demonstrating regions with persistent IEL (large arrow) with marked LP lymphocytosis (small arrow)**.

Over the next 21 months, she continued to have diarrhea despite dietary exclusion therapy, and elevated LFTs (Table 1). Because serum folate and B_12 _remained withing normal limits for the duration of her care, she was not treated with these agents. She did develop a mild iron deficiency anemia, but never required blood transfusions. Endoscopy 15 months after presentation demonstrated progression with grossly friable duodenal mucosa. There was marked IEL with a CD8^+ ^T-cell subset without evidence of enteropathy type T-cell lymphoma (Figure 2). Flow cytometry revealed a nearly 100% T-cell preponderance, the majority of which were a polyclonal αβ T-cell receptor (TCR)^+ ^CD8^+ ^CD7^- ^subset (Table 1: 15 months). Clonality was assessed by polymerase chain reaction of TCR chains. The minority of IELs were CD4^+ ^and CD8^+ ^CD7^+ ^subsets; there were normal numbers of intra-epithelial γδ T-cells and rare B-cells (CD20^+^). Biopsies samples from the stomach and esophagus were normal, as was colonoscopy. There was no evidence of reduced numbers or abnormal morphology of epithelial or goblet cells.

Because of the marked IEL without laboratory or histologic evidence of celiac disease, she was treated with Ciprofloxicin^® ^and Metronidazole^® ^for potential bowel overgrowth and/or tropical sprue. However, six months of antibiotic therapy did not result in clinical or histologic improvement (Table 1: 21 months). Although refractory celiac disease is possible in this scenario, but the lack of elevated intra-epithelial γδ T-cells, normal TTG IgA, and her negative HLA-DQ2/DQ9 genotype make this unlikely. A diagnosis of an immune mediated enteropathy was therefore considered based on the lack of clinical or histologic response to dietary and antibiotic therapy, and the lack of celiac disease serologic markers. Prednisone (60 mg) daily was started.

**Figure 3:**
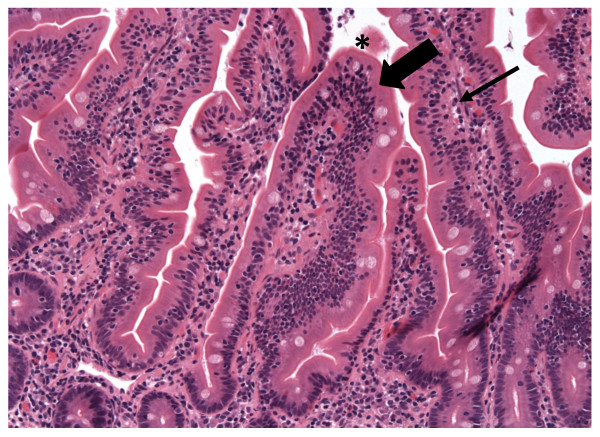
Duodenal biopsy 4 weeks after completing steroid therapy obtained during symptom resolution demonstrating normal tissue (IEL and LP compartments marked by large and small arrows, respectively).

**Figure 4:**
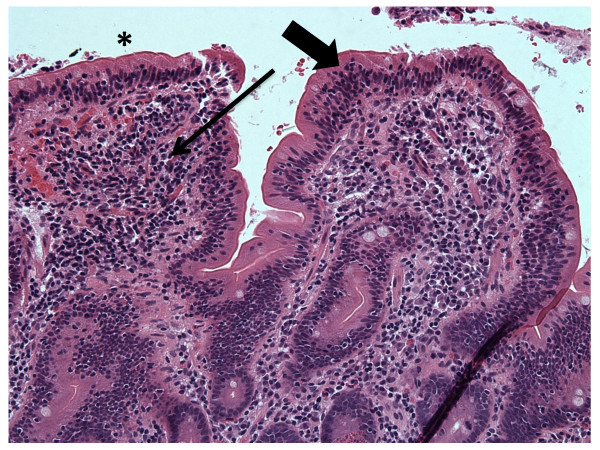
Duodenal biopsy obtained at the same time as in figure 3, but from a different region demonstrating abnormal tissue with blunted architecture (IEL and LP compartments marked by large and small arrows, respectively).

Steroid therapy resulted in marked improvement with symptom resolution accompanied by a 20 lb weight gain. She remained in clinical remission 4 weeks after therapy when repeat Endoscopy demonstrated persistent IEL, but a reduction in the CD8^+^CD7^- ^fraction to 41% of total CD3^+ ^cells (Table 1: 24 months). Histologically, regions with normal villi were now interspersed with abnormal regions (Figures 3 and 4). Her LFTs remained elevated despite the clinical remission and histologic improvement. The patient decided against maintenance therapy with prednisone or azathioprine because she wanted to get pregnant.

### Literature Review

At least 29 cases of AIE have been reported, of which, 26 meet the original diagnostic criteria for AIE [3-9] (Table 2). Eighty five percent (22/26) had anti-epithelial cell antibodies (anti-enterocyte (AEA): n = 17 (65%), anti-goblet cell (AGC): n = 7 (27%), or both: n = 2 (8%)), which were of the IgG class in 76% (13/17) with AEA and 100% (7/7) with AGC. The remaining four patients without antibodies had at least one autoimmune condition (Table 2). Hypothyroidism was most frequently reported, but no disorder was strongly associated with AIE. The majority (69%) had other circulating tissue auto-antibodies, most commonly, ANA (Table 2).

**Table 2:** Reported Cases and Auto-Antibodies

Reference	Gender, Age	Anti-Enterocyte antibodies	Anti- Goblet cell antibodies	Auto-antibodies	Auto-immune involvement	Immune abnormalities
16	F, 38	IgA and IgG	NR	-	NR	NR
16	F, 47	IgA and IgG	NR	Anti-actin Anti-parietal Anti-thyroid microsomal	NR	NR
8	F, 39	+	NR	Anti-thyroid microsomal Anti-thyroglobulin	ITP Raynaud Hepatitis Cholangitis	Impaired antibody production to mitogens and protein antigens
19	F, 50	IgG	NR	-	Perivisceritis Food allergies	NR
18	F, 82	IgG	NR	NR	NR	NR
20	M, 67	NR	NR	NR	NR	NR
20	F, 39	NR	NR	NR	NR	NR
22	F, 58	-	NR	Anti-parietal cell ANCA Anti-thyroglobulin Anti-thyroid peroxidase	T- and B-cell pulmonary infiltrate Autoimmune thyroiditis Sicca syndrome Polyneuropathy Pancreatitis (possible) Autoimmune neutropenia	CD4^+ ^lymphopenia Granulocytopenia
15	M, 54	+	NR	pANCA ANA	NR	NR
3	M, 19	-	IgG	NR	Collagenous enterocolitis Elevated liver enzymes Elevated pancreatic enzymes Atopic dermatitis Uveitis Hyperthyroidism	NR
17	F, 35	IgA and IgG	NR	Anti-CCP ANA	Rheumatoid arthritis	NR
21	M, 59	NR	NR	Anti-intrinsic factor	Pangastritis (atrophic)	NR
4	M, 21	NR	NR	RF ANA	Autoimmune hemolytic anemia Hypothyroidism Raynaud Exfoliative dermatitis	Elevated IgG Low IgA
24	M, 58	+	NR	Anti-parietal cell Anti-thyroid globulin Anti-thyroid peroxidase Anti-adrenal cortical	NR	NR
23	M, 37	IgG	-	ANA	Pancreatic insufficiency	NR
23	M, 75	IgG	IgG	Anti-acetylcholine receptor Anti-striated muscle Anti-glutamic acid dehydrogenase	Myasthenia gravis Thymoma Autoimmune myopathy Autoimmune gastroenterocolitis	NR
23	F, 46	-	IgG	-	Psoriatic arthritis	NR
23	F, 42	-	IgG	-	Atopic dermatitis Autoimmune gastroenterocolitis	NR
23	F, 38	-	IgG	Anti-parietal cell	Pancreatic insufficiency Autoimmune gastroenterocolitis	NR
23	F, 76	IgG	-	ANA	NR	hypogammaglobulinemia (low IgG and low IgM)
23	M, 55	-	-	-	NR	CVID
23	M, 40	IgM	-	ANA	NR	CVID
23	M, 54	-	IgG	-	NR	CVID
23	M, 67	IgA and IgG	-	-	NR	Polyclonal hypergammaglobulinemia
23	F, 45	IgG	IgG	Anti-SSA Anti-phospholipid	Sjogren's RA Raynauds Refractory sprue Lymphocytic colitis	NR
23	M, 59	IgG	-	ANA	Autoimmune gastroenterocolitis	NR
23	M, 75	NR	NR	Anti-saccharomyces cerevisiae antibodies	Autoimmune gastroenterocolotis	Polyclonal hypergammaglobulinemia
23	F, 61	IgG	-	pANCA ANA	Hypothyroidism Refractory sprue	NR
23	F, 59	IgG	-	ANA	Lymphocytic colitis Hypothyroidism	NR

Abbreviations:

ANA: anti-nuclear antibody

ANCA: anti-neutrophil cytoplasmic antibody (p: protoplasmic)

Anti-CCP: *anti*-cyclic citrullinated peptide antibody

CVID: common variable immune deficiency

ITP: idiopathic thrombocytopenic purpura

RF: rheumatoid factor

NR: not reported

Fourteen studies detailed the sites of disease, and 9 provided some immuno-phenotypic analysis (Table 3). The small bowel was the most frequently involved (79% of cases), followed by the colon and stomach. Six (43%) patients had multiple sites and two had extensive disease (stomach, small intestinal and colonic disease) ('generalized autoimmune gut disorder')[5,10,11]. Nine patients (75%) had small bowel IEL at presentation while at least one case reported concurrent colonic IEL. Complete or partial clinical response with therapy was associated with improved IEL in all 8 cases that reported these findings [5-9,11,12]. The IEL was predominantly T-cells (CD3^+^) in at least three cases, and was furthermore CD8^+ ^in at least one of these. T-cell (CD3^+^) lymphocytosis was also reported in the lamina propria in four of five cases that provided this data (Table 3). One case demonstrated extensive abnormalities in CD4^+ ^T-cells, including increased tumor necrosis factor (TNF)α production from activated rectal lymphocytes [5]. However, this finding must be cautiously extrapolated because that patient had active CMV colitis and it is therefore possible that the TNFα response represent epiphenomena.

**Table 3:** Reported Cases with Sites of Disease and Histologic Findings

Reference	Gender, Age	Involved sites	Intra-epithelial	Lamina propria
16	F, 38	Small intestine	46%^a^	NR
16	F, 47	Small intestine	42%^a^	NR
8	F, 39	Stomach, duodenum, jejunum, colon, recto-sigmoid	12%^a ^(normal), decreased CD3^- ^CD103^+^, aberrant CD3^+^CD4^+^CD103^-^, increased HLA-II expression on CD3^+^	Lympho-plasmacytic infiltrate (CD3^+^)
19	F, 50	Duodenum, colon	Duodenum: *IEL = 48/100, CD3^+ ^50/mm (elevated), eosinophils (some);* Colon: *normal*	Colon: *lympho-plasmacytic infiltrate, eosinophils (some)*
18	F, 82	Duodenum	Lympho-plasmacytic infiltrate	NR
20	M, 67	colon	NR	NR
20	F, 39	Small intestine, colon	NR	NR
22	F, 58	Stomach, duodenum, jejunum, colon, rectosigmoid	Duodenum: *IEL = 10-20/100, CD8^+ ^preponderance* *Jejunum: lymphocytosis* Colon: *chronic inflammation, CD4^+^, CD8^+^, CD19^+^/20^+^, CD68^+^*	Duodenum: *CD3+ and CD43+ lymphocytosis with CD68^+ ^(macrophages)* Jejunum: *Inflammatory infiltrate*
15	M, 54	Duodenum, ileum	Duodenum: *IEL = 28/100 (normal), 50% CD8^+^, oligoclonal TCR αβ^+^* Ileum: *lymphocytosis* Colon: *lymphocytosis*	NR
3	M, 19	Duodenum, ileum, colon	Normal	NR
17	F, 35	Duodenum	IEL = > 40/100 52% CD3^+^	NR
21	M, 59	Stomach, colon, ileum	Normal	Colon + Ileum: *lympho-plasmacytic infiltrate, occasional neutrophils and eosinophils* Stomach + Colon + Ileum: *CD8^+ ^and CD45RO^+ ^preponderance*
4	M, 21	Duodenum	IEL = 50/100 CD8^+ ^preponderance	CD4^+ ^preponderance, occasional CD8^+ ^and neutrophils, rare CD20^+^
24	M, 58	Duodenum, ileum, colon	Lymphocytosis	Lympho-plasmacytic infiltrate

Abbreviations:

IEL: Intra-epithelial lymphocytosis

a: expressed as a percentage of total cells

## Discussion

We report a patient with an enteropathy associated with an aberrant CD8^+^CD7^- ^T-cell IEL. Clinically, on histology and by flow cytometry, our patient had characteristic features of an immune mediated enteropathy. The abberant T-cell subset in our patient was reproducible, polyclonal, and correlated with the presence of clinical symptoms. Moreover, the symptom resolution and weight gain with steroid therapy correlated with improvement in villous architecture and a decrease in the CD8^+^CD7^- ^T-cell subset. These facts strongly argue that the T-cell subset was at least partially involved in our patient's enteropathy and was potentially causative, especially considering the failure of dietary exclusion, antibiotics and other therapies in inducing clinical, histologic or immunologic improvement. The salient feature of our case therefore remains the aberrant CD8^+^CD7^- ^IEL in the absence of abnormalities in CD4^+ ^or NK cells as have been previously reported in AIE [5,13].

CD7 is a member of the immuno-globulin superfamily and is expressed on most thymocytes and early in the ontogeny of T- and NK-cells where it is thought to participate in intracellular signaling [14]. The majority of peripheral T-cells in adults bear CD7, but CD7^- ^T-cells are a normally circulating minority comprising 9% of all peripheral blood monocytes (PMBC). The majority of CD7^- ^T-cells are CD4^+ ^(8% of PMBC) with CD8^+^CD7^- ^cells accounting for the remaining 1% of PMBC (CD4^+^/CD8^+ ^ratio = 8)[15]. Although the exact function of CD7^- ^T-cells is uncertain, they appear to be terminally differentiated, activated T-cells. Stimulated CD4^+^CD7^- ^T-cells produce high levels of interleukin-2 and gamma interferon, and compared with CD7^+ ^subsets, have augmented proliferative responses to protein antigens [15]. Furthermore, CD7^- ^T-cells express higher levels of activation markers including CD45RO and CD25 compared with their CD7^+ ^counterparts [15]. CD7^- ^T-cells may also play a role in autoimmune disease evidenced by the increased CD4^+^CD7^- ^cell expansion and accumulation reported in rheumatoid arthritis [16] and psoriasis [17]. These data suggest that the CD8^+^CD7^- ^subset demonstrable in our patient were activated cells that were potentially causative.

The diagnosis of AIE requires the presence of auto-immune phenomena, typically taken to be circulating auto-antibodies to intestinal epithelial cells and/or the presence of auto-immune disease(s) in the absence of celiac disease or PIDs. Our patient had no evidence of anti-epithelial cell antibodies, and in the absence of her diagnosis of AIH, had no other auto-immune conditions. Moreover, her diagnosis of AIH, although based on accepted guidelines, is suspect as she had "auto-antibody negative" AIH, a low pANCA titer, and no liver biopsy [2]. Notably the classical AIE criteria remain controversial, with alternative suggestions to limit the AIE to only those with demonstrable anti-gut epithelial cell antibodies or refine the diagnosis based on histologic immuno-phenotypic characteristics [5]. Regardless of whether our patient meet criteria for AIE, the cumulative evidence suggest our patient had an immune mediated enteropathy. This conclusion is further bolstered by the reduced fraction of CD8^+^CD7^- ^cells concurrent with clinical improvement on prednisone therapy.

Our review finds that AIE is most often characterized by (a) IgG subclass anti-epithelial cell antibodies, (b) preferential small bowel Involvement, and (c) CD3^+ ^αβ TCR^+ ^intra-epithelial and lamina propria lympocytosis. A protean set of histologic abnormalities have been reported, including: villous atrophy, absence of goblet and paneth cells [3,4,18], IEL and infiltrates into the lamina propria (LP). The clinical features and therapeutic response of those with AIE but without anti-epithelial cell antibodies [4,10,19,20] appears indistinguishable to those with antibodies. While the data suggest AIE is associated with a CD3^+ ^T-cell intra-epithelial and lamina propria lymphocytosis, it must be judiciously interpreted due to the paucity of cases that reported immuno-phenotypic findings, and because CD3^+ ^cells are the dominant subtypes in these compartments. CD4^+ ^T cells are thought to be at least partially responsible for AIE based on (a) the phenotypic similarities between AIE and the IPEX syndrome [21,22], (b) the role of CD4^+ ^T cells in villous atrophy [23] and (c) the aberrant CD4^+ ^T cell subsets and CD4^+ ^IEL reported in some cases of AIE [5]. Despite these points, our review suggests that, at present, there is insufficient evidence to definitively confirm or refute this hypothesis in adult onset AIE. It also remains possible that AIE is the common phenotypic result of a heterogeneous set of mechanistic abnormalities rather than a single unified condition.

## Conclusions

Our patient likely had an auto-immune enteropathy associated with an aberrant CD8+CD7- T-cell IEL. Our review of adult onset AIE suggest that the most common features are the presence of IgG type anti-gut epithelial antibodies and preferential small bowel involvement.

## Consent

Written informed consent was obtained and the study was approved by the institutional review board of the University of Kentucky Medical Center.

## Competing interests

The authors declare that they have no competing interests.

## Authors' contributions

SB analyzed the data and drafted the manuscript. VA, DV and RA provided human tissue samples and edited the manuscript (RA). EL analyzed and provided the histologic sections and flow cytometric data. WdW analyzed data and edited the manuscript. All authors have read and approved the final manuscript

## Pre-publication history

The pre-publication history for this paper can be accessed here:

http://www.biomedcentral.com/1471-230X/11/131/prepub
